# Formation and Structure of Highly Ordered Self-Assembled Monolayers on Au(111) via Vapor Deposition of Dioctyl Diselenides

**DOI:** 10.3390/ijms26189192

**Published:** 2025-09-20

**Authors:** Seulki Han, Jin Wook Han, Sicheon Seong, Young Ji Son, Riko Kaizu, Glenn Villena Latag, Tomohiro Hayashi, Jaegeun Noh

**Affiliations:** 1 Department of Chemistry, Hanyang University, 222 Wangsimni-ro, Seongdong-gu, Seoul 04763, Republic of Korea; fhsk0105f@hanmail.net (S.H.); jwhan@hanyang.ac.kr (J.W.H.); ssc09122@hanyang.ac.kr (S.S.); syj2010@hanyang.ac.kr (Y.J.S.); 2 Department of Materials Science and Engineering, School of Materials and Chemical Technology, Institute of Science Tokyo, Yokohama 226-8502, Japan; kaizu.r.aa@m.titech.ac.jp (R.K.); latag.g.aa@m.titech.ac.jp (G.V.L.); 3 Research Institute for Convergence of Basic Science, Hanyang University, 222 Wangsimni-ro, Seongdong-gu, Seoul 04763, Republic of Korea

**Keywords:** self-assembled monolayers, dioctyl diselenide, surface, ordered phase, vapor deposition, scanning tunneling microscopy

## Abstract

The formation and growth processes of octaneselenolate (C8Se) self-assembled monolayers (SAMs) on Au(111) from dioctyl diselenides were investigated by scanning tunneling microscopy (STM) as a function of vapor deposition time at 363 K. STM observations revealed unique surface features of the C8Se SAMs on Au(111) prepared at 363 K for 1 h, consisting of various types of narrow, bright molecular rows and broad, bright molecular rows with three-directional orientations. After increasing the deposition time from 1 and 6 h, interestingly, the structural quality of C8Se SAMs is greatly enhanced, showing the formation of tri-directional highly ordered domains with distinct domain boundaries on an entire Au(111) surface. The observed ordered phase can be described by a (2 × 2√7)rect packing structure with an arial density of 28.9 Å^2^/molecule. C8Se SAMs at 363 K for 24 h consisted of long-range, highly ordered domains and large, disordered domains. The ordered phase can be described by a (√3 × √23)rect packing structure with an arial density of 23.3 Å^2^/molecule, which is denser than that of C8Se SAMs formed by vapor deposition at 363 K and for 6 h. This study clearly demonstrates that vapor deposition is a highly effective method for preparing highly ordered alkyl selenolate SAMs on Au(111). Furthermore, molecular-scale STM results provide new insights into the formation and growth processes of alkyl selenolate SAMs on Au(111) formed by vapor deposition of dialkyl diselenides at high temperatures.

## 1. Introduction

Self-assembled monolayers (SAMs) formed by the spontaneous adsorption of chemically active anchoring groups on solid surfaces are highly attractive and versatile molecular assemblies that can allow for easy control of surface and interface properties [1,2,3,4,5,6]. For example, precise control of surface and interface properties can be readily achieved by varying the anchoring groups, molecular backbone (aliphatic/aromatic), and terminal functional groups of the adsorbed molecules [7,8,9,10,11,12,13,14]. Therefore, SAMs have been widely used in many applications, such as biosensors [2,15], biointerface [2,16], batteries [17], thermoelectric devices [18], solar energy devices [19,20], and molecular electronic devices [1,2,21,22]. Organic thiols are the most widely used precursors for fabricating SAMs on gold surfaces because they can readily form ordered monolayers by forming S-Au bonds through chemical reactions between thiol anchoring groups and the gold surface [1,2,3,5,11,14]. In particular, SAMs formed by alkanethiols are a simple and excellent model system for understanding the molecular self-assembly behavior and structures of organic thiols on metal surfaces [2,3]. Scanning tunneling microscopy (STM) studies revealed molecular-scale features of alkanethiolate SAMs on Au(111) showing various structural phases depending on surface coverage [3,6,23,24,25,26,27]. Alkanethiolate SAMs on Au(111) were found to have a p × √3 structure at low surface coverage, whereas they have the (√3 × √3)R30° or c(4 × 2) structure at saturation coverage [3,6,23,24,25,26,27].

On the other hand, one of the problems with using organic thiols is that they can be oxidized in solution to disulfide or other oxidized groups during SAM formation, which leads to undesirable surface and interface properties of the SAMs [28,29]. To address this issue, new alternatives instead of thiols have been proposed that exhibit high chemical stability in solutions, such as organic thiosulfate [30], thioacetates [28,29,31], and thiocyanates [32,33]. However, SAMs formed by these precursors on gold surfaces generally exhibit inferior structural quality compared to SAMs formed by their corresponding thiol analogues. Additionally, SAMs formed by organic molecules containing selenium atoms, which belong to group 16 of the periodic table, such as sulfur atoms, have attracted considerable attention due to their unique physical, chemical and structural properties [4,12,34,35,36,37,38,39,40,41,42,43,44,45]. Theoretical studies have shown that the adsorption energy of the Se-Au bond in SAMs is stronger than that of the S-Au bond [4,34]. The theoretical results can also be supported by the fact that the reductive desorption of aliphatic or aromatic selenolate SAMs on Au electrodes occurs at a higher negative potential than that of thiolate SAMs [35]. Thermal desorption spectroscopy (TDS) measurements revealed that the main thermal desorption peak of CH_3_Se species in SAMs Au(111) surfaces appeared at 416 K via the cleavage of CH_3_Se–Au bonds, whereas that of CH_3_S species appeared at around 366 K via the cleavage of CH_3_S–Au bonds [36].

Surface structure, work function, and conductance of selenolate SAMs are significantly different from those of thiolate SAMs [37,38,39,40,41,42,43,44,45]. Interestingly, selenolate SAM-based molecular electronic devices were found to be much more stable and have much longer lifetimes than thiolate-based electronic devices, due to the formation of strong Se-Au bonds [37]. Molecular-scale STM studies revealed that the formation and structure of aliphatic and aromatic selenolate SAMs on Au(111) are also significantly different from those of thiolate SAMs with the same molecular backbone [40,41,42,43,44,45]. Aromatic and alicyclic selenolate SAMs on Au(111) have larger ordered domains with a few structural defects compared to the corresponding thiolate SAMs [41,42,43]. Meanwhile, although many STM studies for alkanethiolate SAMs on Au(111) have been reported to understand the molecular-scale surface properties of SAMs depending on the alkyl chain length and experimental conditions [2,3,6,23,24,25,26,27,46,47,48,49], few STM studies for alkaneselenolate SAMs formed from dialkyl diselenides have been reported [44,45]. STM observations revealed that dodecaneselenolate SAMs on Au(111) consist of closely packed local ordered domains exhibiting moiré patterns and linear missing-row domains, which have never been observed in alkanethiolate SAMs [44]. When typical SAM preparation conditions (1 mM concentration, room temperature, and 24 h deposition) were used, closely packed and well-ordered alkanethiolate SAMs exhibiting a c(4 × 2) structure were usually formed, whereas alkaneselenolate SAMs had only disordered phase [45]. On the other hand, loosely packed ordered SAMs with many structural defects were formed when diluted μM solutions and/or very short immersion times of less than 1 h were used [44,45]. It has been demonstrated that the formation and structure of ordered domains in alkanethiolate SAMs are significantly influenced by solvent properties [2,3,26,33,50]. It was also reported that the structural quality of alkaneselenolate SAMs on gold can be largely improved when formed from neat liquid of dialkyl diselenides, whereas less ordered SAMs were obtained when SAMs were formed in solutions, which means that solvent molecules significantly hinder the formation of selenolate SAMs [50]. Furthermore, it has been reported that the structural quality of alkaneselenolate SAMs on gold can be significantly improved when formed from a pure liquid of dialkyl diselenides, whereas less ordered SAMs are obtained when formed from solution [51]. Adsorption of octyl selenocyanates on Au(111) in a 1 mM ethanol solution resulted in poorly ordered SAMs, whereas vapor deposition resulted in the formation of highly ordered octyl selenocyanate SAMs [52]. These results suggest that solvent molecules significantly hinder the formation of selenolate SAMs. Therefore, we believe that vapor deposition technique is very effective in eliminating the negative side effects of solvents on the formation and structure of alkyl selenolate SAMs, as demonstrated in the formation of alkanethiolate or alkyl selenocyanate SAMs [52,53].

**Figure 1:**
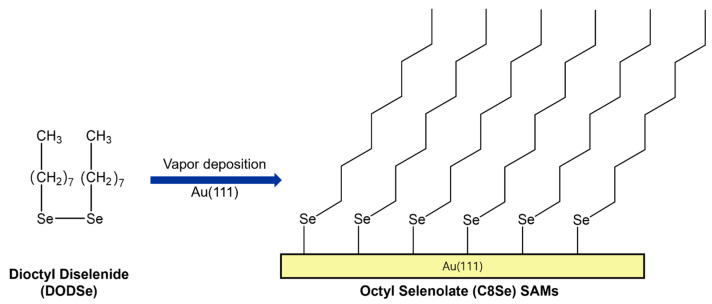
Chemical structure of dioctyl diselenide (DODSe) and the formation of C8 Se SAMs on Au(111) by the adsorption of DODSe molecules from ambient-pressure vapor deposition.

In this study, to extend and deepen the understanding of the formation and structure of alkyl selenolate SAMs on Au(111) by vapor deposition of dialkyl diselenides, the surface structure of SAMs of dioctyl diselenide [DODSe, C8Se–SeC8] formed on Au(111) at high temperatures and for various vapor deposition times was investigated using STM. Figure 1 shows the chemical structure of DODSe and the formation of octaneselenolate (C8Se) SAMs by vapor adsorption of DODSe on Au(111). Herein we report the molecular-scale STM results demonstrating the formation of highly ordered C8Se SAMs on Au(111) with a (2 × 2√7)rect packing structure by vapor deposition at 363 K for 6 h. Furthermore, this study clearly demonstrates that vapor deposition is a highly effective method for preparing well-ordered alkyl selenolate SAMs compared to solution deposition.

## 2. Results and Discussion

### 2.1. Formation of Short-Range Ordered C8Se SAMs on Au(111) from Vapor Deposition of DODSe at 363 K for 1 h

**Figure 2:**
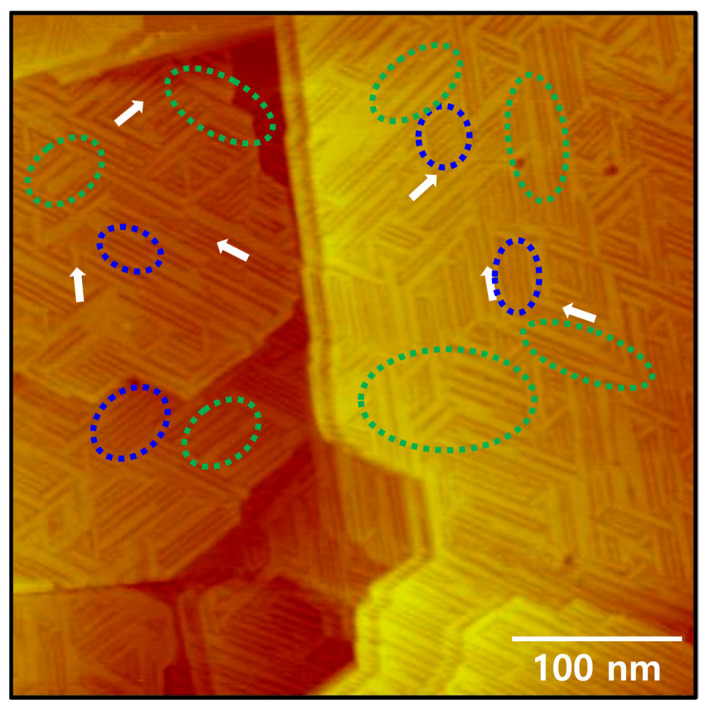
Large-scale STM image (400 nm × 400 nm) showing the unique surface features of C8Se SAMs on Au(111) formed by vapor deposition at 363 K for 1 h. Tri-directional domain orientations are indicated by white arrows. Narrow and wide rows are indicated by blue and green dotted ovals.

**Figure 3:**
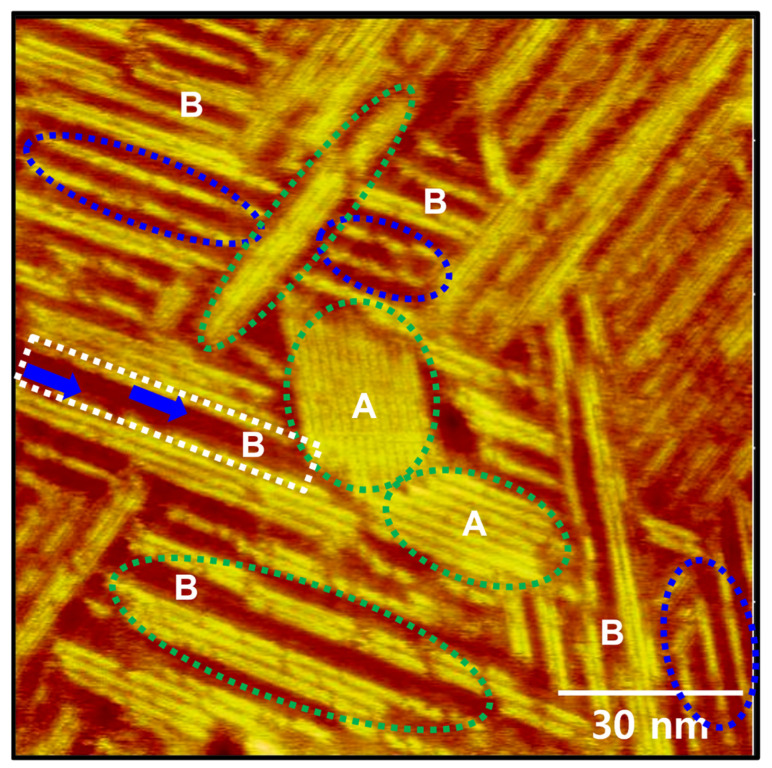
Enlarged STM image (120 nm × 120 nm) showing various molecular rows with tri-directional orientations for C8Se SAMs on Au(111) formed by vapor deposition at 363 K for 1 h. The short-range ordered domains are indicated by A, and the dark areas between the bright ordered rows are indicated by B. Single and multiple molecular rows are indicated by blue and green dotted ovals, respectively. Molecular rows in the dark area are indicated by blue arrows.

Spectroscopic measurements revealed that alkanethiolate (RS^−^, R = alkyl group) or alkaneselenolate (RSe^−^) SAMs on gold surfaces can be formed via dissociative adsorption of S–S bond cleavage of dialkyl disulfides [2,3,4,36,54] or Se–Se bond cleavage of dialkyl diselenides [24,36,44,55]. Theoretical studies also support experimental results showing that organic selenium molecules react with gold surfaces to form SAMs through the formation of Se-Au bonds [4,34]. Therefore, as demonstrated in previous many studies [2,3,4,44,45], it is reasonable to expect that the adsorption of DODSe (C8Se–SeC8) on Au(111) results in the formation of C8Se SAMs, as shown in Figure 1. Note that our reductive desorption measurements showed that C8Se SAMs were formed through chemical bond formation (Se–Au bond) between DODSe and the Au(111) surface via vapor deposition. Figure 2 shows STM image presenting the unique surface features of C8Se SAMs on Au(111) prepared via vapor deposition at 363 K for 1 h. Interestingly, large-scale STM image (400 nm × 400 nm) shows that several Au(111) terraces are completely covered by heterogeneous rows as a result of the formation of C8Se SAMs. The resulting SAMs consist of various types of narrow, bright rows (e.g., indicated by blue dotted ovals) and wide, bright rows (e.g., indicated by green dotted ovals), which have not been observed in other alkanethiolate or alkaneselenolate SAMs to date. In contrast, uniform rows with identical interrow distances were frequently observed over the entire Au(111) surface during the growth stage of alkanethiolate SAMs [6,23,46] or alkyl selenolate SAMs formed in ethanol solution [45]. The structural details will be discussed in Figure 3. In addition, tri-directional domain orientations with domain angles of 60° or 120° were observed on various Au terraces, as indicated by the white arrows in the STM image of Figure 2. The observed domain orientations closely follow the three-fold symmetry of the Au(111) lattice, suggesting a strong commensurable relationship between the C8Se SAM structure and the Au(111) lattice. Therefore, the formation of the C8Se SAMs is believed to be primarily driven by the chemical interactions between the Se anchoring groups and the Au(111) surface. Similar commensurable SAM structures have also been observed for alkanethiolate SAMs [25,26] alicyclic selenolate SAMs [43], and alkyl selenocyanate SAMs [52]. Previous works have also formalized that the formation of commensurable or incommensurable structures for adlayers on solid substrates can be determined by competitive interactions between molecule–molecule and molecule–substrate [56].

Enlarged STM image (120 nm × 120 nm) in Figure 3 shows that various types of ordered molecular rows for C8Se SAMs on Au(111) formed by vapor deposition at 363 K for 1 h. Figure 3 clearly reveals that the narrow, bright ordered rows in STM image of Figure 2 usually consist of single molecular rows (indicated by blue dotted ovals), whereas the wide, bright ordered rows consist of multiple molecular rows (indicated by green dotted ovals). In particular, the short-range ordered domains, labeled A, were formed after many molecular rows coalesced due to the growth of SAMs. Dark regions between the bright ordered rows were also observed, labeled B. The height difference between the ordered rows and the dark region B was found to be less than 1 Å, which is much smaller than molecular length of C8Se, 14.3 Å [45] or vacancy islands with a depth of 2.5 Å that often appeared from thioate or selenolate SAMs [2,3,4,5,6,7,25,26,33,36,43,52,53]. This result means that the dark region is not bare gold surface or vacancy islands. High-resolution STM image reveals the presence of molecular rows in the dark region indicated by blue arrows (within the white dotted rectangular box). Therefore, it is assumed that the octyl chains in C8Se SAMs in the dark region are more inclined toward the Au(111) surface, suggesting that the surface coverage in the dark region is lower than that in the bright region. This suggestion can be supported by the fact that loosely packed thiolate monolayers show darker imaging contrast than densely packed monolayers in STM observations [23,48,57]. Similar results showing a mixed phase containing dark and bright ordered domains were observed for octyl selenocyanate SAMs on Au(111) formed via vapor deposition 348 K for 6 h [52]. Therefore, the presence of these inhomogeneous surface features of C8Se SAMs formed at 363 K for 1 h strongly suggests that the C8Se SAMs have not yet reached a thermodynamically stable phase, since densely packed thiolate or selenolate SAMs on Au(111) have a very uniform surface showing long-range ordered domains [25,26,43,52]. On the other hand, despite numerous attempts to obtain molecular-level surface structures of C8Se SAMs formed at 363 K for 1 h, observing individual molecules in the molecular rows is extremely difficult. This is likely due to the lack of crystallinity of the C8Se SAMs, which can be supported by the presence of various structural phases as shown in Figure 3.

### 2.2. Formation of Highly Ordered C8Se SAMs on Au(111) from Vapor Deposition of DODSe at 363 K for 6 h

**Figure 4:**
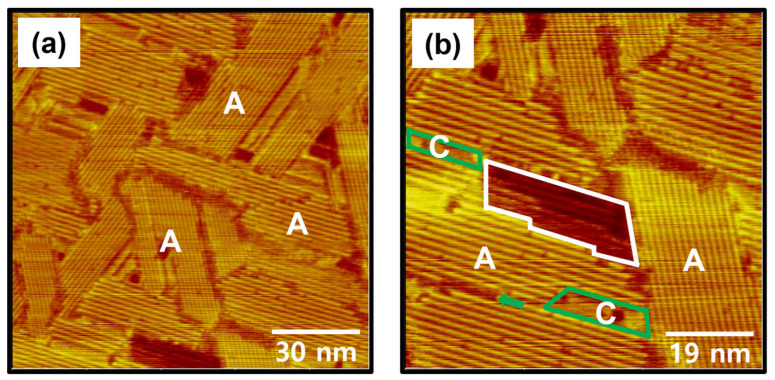
STM images showing well-ordered domains of C8Se SAMs on Au(111) formed by vapor deposition at 363 K for 6 h. The scan sizes of the STM images are (**a**) 120 nm × 120 nm and (**b**) 76 nm × 76 nm. Ordered and disordered domains are indicated by A and C (green solid lines). Vacancy islands are indicated by white solid lines.

Fabrication of uniform SAMs with closely packed and long-range ordered phases is very important issue because it can provide highly reproducible physical and chemical properties of SAM-based devices [1,2,3,9,21,58]. Therefore, optimization of SAM fabrication conditions such as deposition method, deposition time and temperature is an important factor in obtaining highly ordered SAMs with a uniform surface [3,5,7,24,42,43,44,45,52]. To understand the effect of deposition time on the formation of C8Se SAMs on Au(111), the deposition time was increased from 1 h to 6 h at 363 K. The STM images in Figure 4 show the surface structures of C8Se SAMs on Au(111) formed at 363 K for 6 h. Molecular-scale STM observations clearly revealed a significant structural change in C8Se SAMs on Au(111) from domains with non-uniform molecular rows (Figure 3) to domains with uniform molecular rows (Figure 4) with increasing deposition time. STM images in Figure 4a,b show the formation of tri-directional well-ordered domains (labeled A) for C8Se SAMs with distinct domain boundaries after a longer deposition of 6 h. This is attributed to the enhanced van der Waals interactions between the octyl chains of the C8Se SAMs and the increased chemical interactions between the Se anchoring groups and the Au(111) surface as the surface coverage increased. After sufficient deposition time, highly ordered SAMs with clear domain boundaries have also been observed in various other thiolate and selenolate SAMs, although the molecular packing structures are different [3,11,25,26,43,52,53]. Meanwhile, the size of the well-ordered domains of C8Se SAMs was observed to be in the range of approximately 30 nm to 60 nm. The dark area indicated by white solid lines in Figure 4b corresponds to vacancy island typically observed in well-ordered thiolate or selenolate SAMs chemically adsorbed on the Au(111) surface [3,4,5,25,26,40,43,44]. Therefore, the presence of vacancy islands in C8Se SAMs provides clear evidence that these SAMs prepared by vapor deposition are formed through chemical interactions between the Se anchoring groups and the Au(111) surface. The detailed formation mechanism of vacancy islands was described in previous literature [25]. STM observations revealed that the vacancy islands were also covered with ordered molecular rows, indicating that the vacancy islands were not bare gold surfaces as demonstrated in other studies [25,43]. On the other hand, some structural defects were also observed such as missing rows (indicated by green arrows) or disordered phases (indicated by green solid lines, labeled C) in the ordered domains (Figure 4b). It should be noted that alkyl selenolate SAMs on Au(111) formed by adsorption of dialkyl diselenides or alkyl selenocyanates from solution have poorly ordered phase or non-uniform complicated structural phases containing densely packed distorted and missing row structures [44,45,52]. In contrast, the present study clearly demonstrated that highly ordered alkyl selenolate SAMs can be prepared by vapor deposition at 363 K by controlling the deposition time.

**Figure 5:**
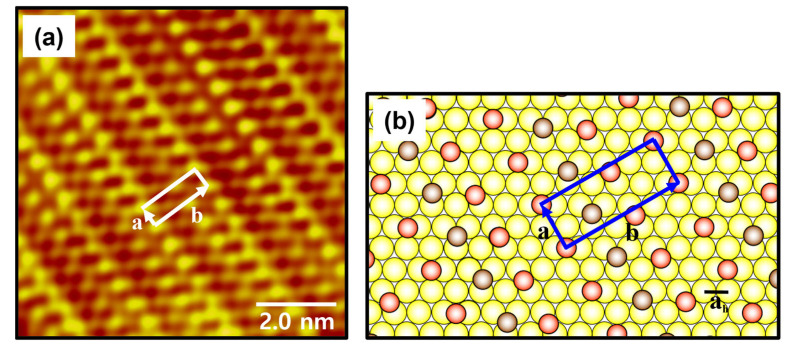
(**a**) A low-pass filtered high-resolution STM image (8 nm × 8 nm) of C8Se SAMs on Au(111) formed by vapor deposition at 363 K for 6 h. (**b**) A proposed structural model of C8Se SAMs on Au(111). The red and peanut-colored circles correspond to C8Se molecules, and the yellow circles correspond to Au atoms in the structural model. The unit cell is indicated by a white and blue square, and the unit cell vectors a and b are indicated by white and blue arrows in the STM image and structural model.

Figure 5a shows a low-pass filtered, high-resolution STM image (8 nm × 8 nm) of the ordered phase of C8Se SAMs on Au(111) formed at 363 K for 6 h. The ordered phase consists of one dark and two bright molecular rows arranged alternately within the unit cell. Based on the STM observations, the lattice constants of a rectangular unit cell containing three molecules were extracted as follows: a = 5.8 ± 0.2 Å = 2a_h_ and b = 15.5 ± 0.2 Å = 2√7a_h_, where a_h_ = 2.89 Å corresponds to the interatomic distance of gold atoms. A structural model of the ordered phase for C8Se SAMs on Au(111) was proposed in Figure 5b. The ordered phase can be described by a (2 × 2√7)rect packing structure. In this model, it is proposed that the selenium atoms in the bright molecular rows occupy the bridge sites of the Au(111) lattice, and the selenium atoms in the dark molecular rows occupy the three-fold hollow sites. The occupied volume per adsorbed molecule was calculated to be 28.9 Å^2^/molecule, which is higher than the 21.6 Å^2^/molecule of closely packed octanethiol (C8S) SAMs on Au(111) with the commonly observed (√3 × √3)R30° or a c(4 × 2) structure [6,26,47]. This result means that highly ordered C8Se SAMs are 1.34 times more loosely packed than the closely packed alkanethiolate SAMs.

### 2.3. Coexistence of Long-Range Ordered and Disordered Phases of C8Se SAMs on Au(111) from Vapor Deposition of DODSe at 363 K for 24 h

**Figure 6:**
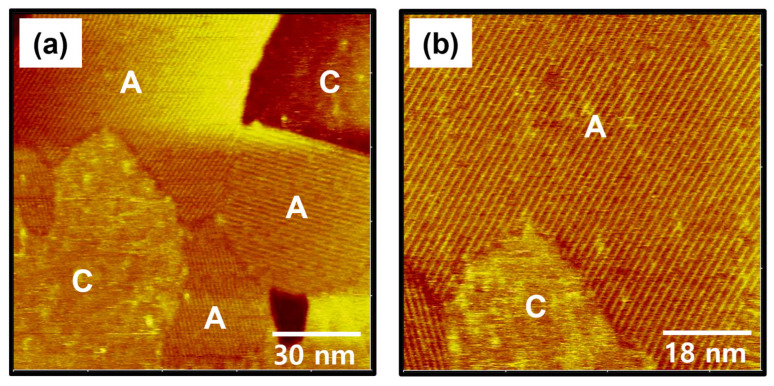
STM images showing the mixed phase of C8Se SAMs on Au(111) formed by vapor deposition at 363 K for 24 h. The scan sizes of the STM images are (**a**) 120 nm × 120 nm and (**b**) 72 nm × 72 nm. Ordered and disordered domains are indicated by A and C.

STM observations in this study revealed that the structural order of C8Se SAMs on Au(111) significantly improved as the deposition time increased from 1 to 6 h. Therefore, understanding the surface properties of C8Se SAMs after long-term deposition is very important from the perspective of obtaining information that can control the structure and properties of C8Se SAMs. STM images in Figure 6 show the formation and surface structures of C8Se SAMs on Au(111) formed after long-term vapor deposition at 363 K for 24 h. Interestingly, C8Se SAMs were composed of long-range, well-ordered domains (labeled A) and a large, disordered phase (labeled C), which are quite different from those observed in C8Se SAMs formed at 363 K for 6 h, as shown in Figure 4. After long-term deposition for 24 h, notable structural changes were observed: (i) the formation of large-sized ordered domains by coalescence of small-sized ordered domains through the Ostwald ripening processes at high temperature [59,60,61] and (ii) the formation of large-sized disordered domains as a result of preferential desorption of C8Se molecules from the SAMs by longer deposition time at a higher temperature of 363 K. This suggestion may be supported by the observation that loosely packed decanethiol SAMs were formed through desorption of adsorbed molecules from closely packed, ordered domains during prolonged deposition in solution at 348 K for 20 h [49]. Our STM studies clearly demonstrated that after prolonged deposition for 24 h, the size of the ordered domains in the C8Se SAMs increased, but so did the disordered phase. Therefore, optimizing the appropriate deposition conditions is crucial for fabricating large-area, well-ordered C8Se SAMs. In particular, it was found that highly ordered C8Se SAMs on Au(111) with high structural quality could be fabricated after deposition at 363 K for 6 h.

**Figure 7:**
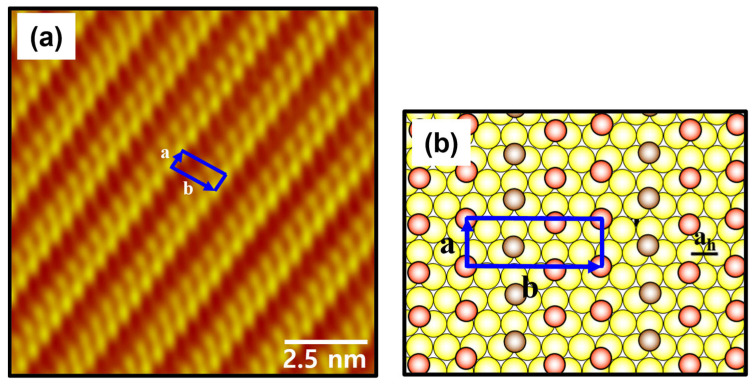
(**a**) A low-pass filtered high-resolution STM image (10 nm × 10 nm) of C8Se SAMs on Au(111) formed by vapor deposition at 363 K for 24 h. (**b**) A proposed structural model of C8Se SAMs on Au(111). The red and peanut-colored circles correspond to C8Se molecules, and the yellow circles correspond to Au atoms in the structural model. The unit cell is indicated by a blue square, and the unit cell vectors a and b are indicated in the STM image and structural model.

The high-resolution STM image (10 nm × 10 nm) in Figure 7a shows a well-ordered phase of C8Se SAMs on Au(111) formed by vapor deposition at 363 K for 24 h. Based on the STM observations, the lattice constants for a rectangular unit cell containing three molecules were deduced as follows: a = 5.0 ± 0.2 Å = √3a_h_ and b = 14.1 ± 0.2 Å = √23a_h_, where a_h_ = 2.89 Å is the distance between gold atoms. The structural model of this ordered phase of C8Se SAMs on Au(111) is shown in Figure 7b and the ordered phase can be represented by a (√3 × √23)rect packing structure. It can be predicted that the selenium atoms in the SAMs adsorb the bridges and triple-hollow structures of the Au(111) lattice. The occupied volume per adsorbed molecule was calculated to be 23.3 Å^2^/molecule, which is denser compared to the (2 × 2√7)rect structure of C8Se SAMs with 28.9 Å^2^/molecule (deposition at 363 K and for 6 h) and very closed to that of 21.6 Å^2^/molecule for C8S SAMs with a c(4 × 2) superlattice. The surface coverage of the ordered domains of C8Se SAMs was found to increase as the deposition time increased from 6 to 24 h. Therefore, it is reasonable to assume that the main driving force for the structural transitions of C8Se SAMs on Au(111) from the (2 × 2√7)rect to the (√3 × √23)rect structure is the increased surface coverage due to longer deposition for 24 h.

## 3. Materials and Methods

### 3.1. Chemicals and Preparation of Au(111) Substrates

DODSe was synthesized by refluxing Na_2_Se_2_ and 1-bromooctane in methanol for 3 h according to a previously described method [62]. The product was confirmed by ^1^H-NMR (400 MHz, CDCl_3_): 0.866–0.901 (t, 6H), 1.280 (s, 16H), 1.349–1.401 (m, 4H), 1.689–1.762 (m, 4H), 2.894–2.932 (t, 4H). The single-crystal Au(111) substrates for SAM preparation were fabricated by thermal evaporation of gold onto freshly cleaved mica sheets preheated at 623 K in an ultrahigh vacuum condition of approximately 10^−5^ Pa [52]. STM measurements clearly show that the Au(111) substrates have an intrinsic herringbone structure, which is observed on clean Au(111) surfaces as previously reported [63].

### 3.2. Preparation of C8Se SAMs from DODSe

C8 Se SAMs were fabricated by placing clean Au(111) substrates in 3 mL V-vials containing 2 μL of pure DODSe liquid as a function of vapor deposition time at 363 K. The V-vials were then tightly sealed with caps and maintained at 363 K for 1, 6, and 24 h, respectively. When placing the Au(111) substrate into the V-vials, care must be taken to avoid direct contact with the pure liquid. The fabricated SAM samples were thoroughly washed with pure ethanol to remove the DODSe molecules physisorbed on the SAM surface, and then dried in a high-purity N_2_ gas flow.

### 3.3. STM Measurements

STM measurements were performed using NanoScope E (Veeco, Santa Barbara, CA, USA) using commercially available Pt/Ir (80:20) tips. STM images were acquired using constant current mode in air at RT. For STM observation, a bias voltage (*V_b_*) of 200 to 500 mV and a tunneling current (*I_t_*)of 0.30 and 0.70 nA were applied between the tip and the sample.

## 4. Conclusions

The formation and growth processes of C8Se SAMs on Au(111) were investigated by STM as a function of vapor deposition time at 363 K. Interestingly, STM observations revealed unique surface features of C8Se SAMs on Au(111) prepared via vapor deposition at 363 K for 1 h, including various types of narrow, bright rows and wide, bright molecular rows. After increasing deposition time from 1 to 6 h, molecular-scale STM observations clearly revealed a significant structural change in C8Se SAMs showing the formation of tri-directional well-ordered domains with distinct domain boundaries on entire Au(111) surfaces. The ordered phase can be described by a (2 × 2√7)rect packing structure with an average arial density of 28.9 Å^2^/molecule. After long-term deposition at 363 K for 24 h, C8Se SAMs were composed of long-range, well-ordered domains and large, disordered domains, which are quite different from those observed in C8Se SAMs formed at 363 K for 6 h. The ordered phase can be described by a (√3 × √23)rect packing structure with an average arial density of 23.3 Å^2^/molecule, which is denser than that of C8Se SAMs formed by vapor deposition at 363 K and for 6 h. In this study, we clearly demonstrated that highly ordered alkyl selenolate SAMs can be prepared by vapor deposition method at 363 K by controlling the deposition time. We believe that our results will be very useful for fabricating SAM-based electronic devices with highly reproducible performance. Moreover, we can provide very useful information for understanding the formation and surface structures of alkyl selenolate SAMs on Au(111) formed via vapor deposition of dialkyl disulfides at high temperatures.

## Data Availability

The data presented in this study are available in this article.
